# Mitochondrial Dysfunction in Kidney Disease and Uremic Sarcopenia

**DOI:** 10.3389/fphys.2020.565023

**Published:** 2020-09-04

**Authors:** Koji Takemura, Hiroshi Nishi, Reiko Inagi

**Affiliations:** ^1^Division of Nephrology and Endocrinology, Graduate School of Medicine, The University of Tokyo, Tokyo, Japan; ^2^Division of CKD Pathophysiology, Graduate School of Medicine, The University of Tokyo, Tokyo, Japan

**Keywords:** mitochondria, CKD - chronic kidney disease, sarcopenia, kidney, skeletal muscle

## Abstract

Recently, there has been an increased focus on the influences of mitochondrial dysfunction on various pathologies. Mitochondria are major intracellular organelles with a variety of critical roles, such as adenosine triphosphate production, metabolic modulation, generation of reactive oxygen species, maintenance of intracellular calcium homeostasis, and the regulation of apoptosis. Moreover, mitochondria are attracting attention as a therapeutic target in several diseases. Additionally, a lot of existing agents have been found to have pharmacological effects on mitochondria. This review provides an overview of the mitochondrial change in the kidney and skeletal muscle, which is often complicated with sarcopenia and chronic kidney disease (CKD). Furthermore, the pharmacological effects of therapeutics for CKD on mitochondria are explored.

## Introduction

**FIGURE 1:**
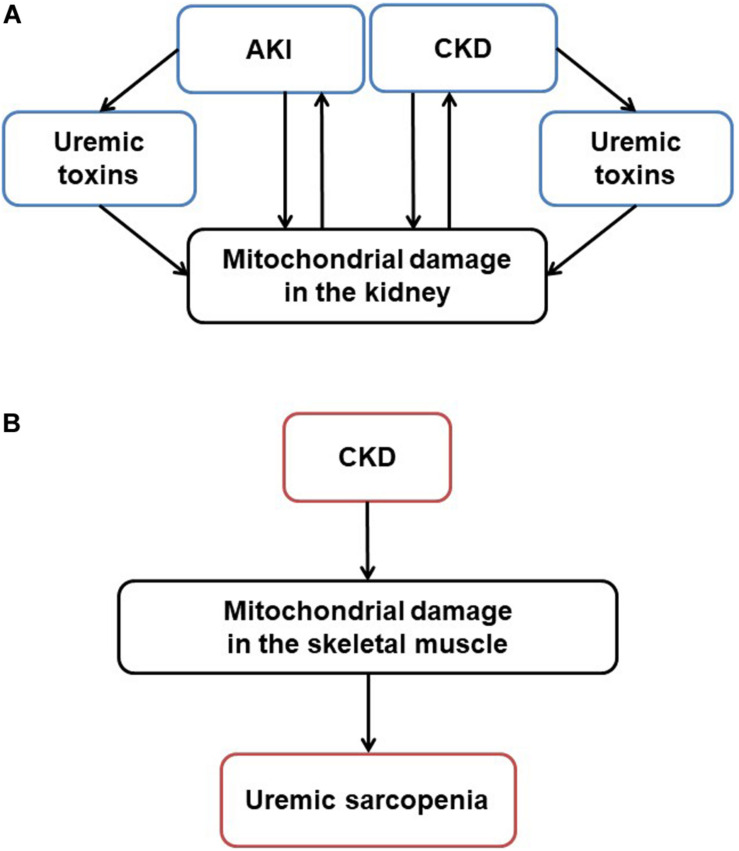
Mitochondrial damage in CKD and uremic sarcopenia. **(A)** Stressors like sepsis, ischemia and toxins induce acute mitochondrial damage. Pathogenesis such as hypertension, diabetes, and obesity induce chronic mitochondrial damage. Uremic toxins accumulated in AKI and CKD also induce mitochondrial damage. On the other hand, mitochondrial damage itself exacerbates kidney damage, forming a vicious cycle in CKD progression. **(B)** In CKD, mitochondrial damage mediates uremic sarcopenia. AKI, acute kidney injury; CKD, chronic kidney disease; ROS, reactive oxygen species; ATP, adenosine triphosphate.

Mitochondria originated approximately 1.5 billion years ago from α-proteobacterium via symbiosis within an ancestral eukaryotic host cell. Although mitochondria contain double membrane and serve as the main producer of adenosine triphosphate (ATP), their form and composition have evolved, and these organelles have gained a myriad of additional functions. This article reviews mitochondrial functions, the changes of these organelles in the kidney (Figure 1A) and skeletal muscle tissues (Figure 1B) in kidney diseases, and the potential effects of therapeutic agents on the mitochondria in treating kidney diseases and uremic sarcopenia.

## Mitochondria Biology in the Kidney

### Mitochondrial Structure

Mitochondria are intracellular organelles found in all eukaryotes. The mitochondrial structure contains a membrane structure of outer and inner mitochondria membrane (IMM) layers, with one compartment between the intermembrane space and the inner matrix. The mitochondrial outer mitochondria membrane (OMM) contains a porin that controls the transport of proteins into mitochondria and allows non-selective permeation of small molecule substances of approximately 1,550 kDa. The IMM contains a complex folded structure, the cristae, that contributes to oxidative phosphorylation to produce ATP, which is an energy source in the cell.

The renal proximal tubules contain more mitochondria than any other compartments in the kidney. Renal proximal tubules absorb more than 65% of the filtrate that passes through the glomerular membrane filter, such as glucose, ions, and albumin (Rector, 1983). Mitochondria often undergo transformation in both physiological and pathological conditions (Wakabayashi, 2002). In a diseased kidney, premorse and gigantic mitochondria are noted in the cytoplasm of the proximal epithelial cells, as shown in early studies of human kidney samples using electron microscopy (Suzuki et al., 1975). The experimental literature also supports that mitochondria amounts largely diminish and their structure appears altered after acute kidney injury (AKI) (Lan et al., 2016). Although the molecular mechanism underlying the morphologic alteration has yet to be investigated, mitochondria represent one of the more vulnerable organelles for various types of toxic and pathogenic insults.

### Mitochondrial Chromosome, Genome, DNA

#### Mitochondrial Biogenesis

The mitochondrial mass is increased through the cellular process called mitochondrial biogenesis to adapt to the ever-changing energy demand. Mitochondrial biogenesis leads to a greater mitochondrial metabolic capacity by increasing synthesis of metabolic enzymes. While the majority of mitochondrial molecules are encoded by the cell nuclear genes, most parts of the electron transport chain (ETC) that function as an energy generator are produced from mitochondrial genes. Mitochondrial DNA (mtDNA) is transcribed by the mitochondrial DNA-directed RNA polymerase, POLRMT (Graziewicz et al., 2006) and the essential enhancer is the mitochondrial transcription factor A (TFAM), which ensures mtRNA unwinding and flexing required for the POLRMT binding to the promoters. The expression of TFAM is regulated by nuclear respiratory factor 1 (Nrf1) binding to the specific promoter sites (Virbasius and Scarpulla, 1994; Gureev et al., 2019), suggesting that Nrf1 may be involved the biogenesis and energy production in the mitochondria (van Tienen et al., 2010; Benner et al., 2013). Also, researchers have discovered that peroxisome proliferator-activated receptor γ coactivator 1α (PGC-1α) in brown adipocytes is a contributing factor for cold-mediated mitochondrial biogenesis (Puigserver et al., 1998). This transcription coactivator promotes the transcription of Nrf1 although PGC-1α acts as a coactivator for numerous genes including Nrf2 (Wu et al., 1999). PGC-1α also activates mitochondrial biogenesis in skeletal muscle and enhances the slow-twitch manifestations of the skeletal muscle, such as fatty acid oxidation and an increase in type I myosin heavy chain (Wu et al., 1999; Jornayvaz and Shulman, 2010). Furthermore, this coactivator is important for skeletal muscle remodeling in physical exercise (Lira et al., 2010).

In sepsis-associated AKI, the mitochondrial function deteriorates, and the expression of genes involved in oxidative phosphorylation is reduced (Parikh et al., 2015). Particularly, PGC-1α expression decreases as renal function declines, and renal function was impaired due to prolonged sepsis. The activation of PGC-1α may promote recovery from AKI caused by sepsis, and this application in therapy is expected. Recently, our laboratory revealed that mtDNA copy number and PGC-1α expression were reduced in the kidneys of animals with polycystic kidney disease (Ishimoto et al., 2017). Moreover, the eradication of mitochondrion-specific oxidants reduced intracellular superoxide and halted the proliferation of cyst epithelial cells via extracellular signal-related kinase inactivation (Ishimoto et al., 2017).

#### Mitochondrial DNA Leakage

In cells infected with pathogens (e.g., DNA viruses), the pathogen-derived double-stranded DNA appears in the cytoplasm. Cyclic GMP–AMP synthase (cGAS) is a pattern recognition receptor that recognizes double-stranded DNA in the cytoplasm and then binds to the trans-membrane protein, a stimulator of interferon genes (STING) localized on the endoplasmic reticulum (ER). Eventually, this reaction induces a type I interferon-mediated host defense response to DNA viruses (Chen et al., 2016). Moreover, the cGAS–STING pathway activation is involved in autoimmune and inflammatory reactions, likely resulting from the activation of cGAS by self-genomic DNA damage (Li and Chen, 2018).

Our group recently clarified the relationship between mitochondrial damage and the induction of cGAS–STING pathway in inflamed proximal tubular cells. In cisplatin-induced AKI, the cGAS–STING pathway was activated in the kidney (Maekawa et al., 2019). In STING-deficient mice, cisplatin-induced renal dysfunction and inflammatory responses were reduced. Additionally, the mitochondrial membrane potential was reduced in renal proximal tubular cells stimulated with cisplatin, and mtDNA leaked into the cytosol, thereby causing the activation of the cGAS–STING pathway (Maekawa et al., 2019). The inhibition of this pathway is a promising target for future treatments of AKI.

### Mitochondrial Dynamics

Although mitochondrial biogenesis promotes new mitochondria production, mitochondria cannot be generated *de novo*. Instead, mitochondria form a dynamic network that can alter the shape and size and also add new content to pre-existing mitochondria. In other words, mitochondria actively and frequently undergo fusion and division (fission), such that long and extended structures are formed through fusion, and small fragmentation is induced through the division. Mitochondrial fusion is caused by several guanosine triphosphate hydrolases (GTPase), including mitofusin 1 (Mfn1), Mfn2, and optic atrophy 1 (Opa1). Both Mfn1 and Mfn2 are considered to contribute to OMM fusion, whereas Opa1 splicing contributes to IMM fusion. Conversely, mitochondrial fission is promoted by fission 1 protein (Fis1), that is localized on the OMM, and the GTPase, dynamin-related protein (Drp1). The mutation of Drp1 is associated with lethal neonatal defects in humans (Waterham et al., 2007), and the mice deficient in Drp1 are also embryonic lethal, whereas brain-specific Drp1 ablation causes developmental defects to the cerebellum (Wakabayashi et al., 2009). These findings suggest that mitochondrial fission plays an essential role in early development and differentiation.

In ischemic and cisplatin nephrotoxic AKI, these mitochondrial dynamics have been analyzed mainly in proximal tubules that are dependent on oxidative phosphorylation for the large demand of ATP necessary for solute transportation. Mitochondrial fission initiated by Drp1 translocation to the OMM is often observable immediately after the injury (Brooks et al., 2009). Intriguingly, treatment with a small molecule that inhibits Drp1, Mdivi1, attenuated both tubular cell apoptosis and tubular tissue damage. Also, in cultured tubular cells, the silencing of *Drp1* or a dominant-negative Drp1 induction led to mitochondrial fragmentation and subsequent apoptosis. This concept of Drp1 to mediate not only mitochondrial fission but also subsequent ischemic renal tissue damage was further supported by the murine genetic deletion of *Drp1* in the proximal tubule epithelium (Perry et al., 2018). They also showed that delayed deletion of Drp1 in the recovery period after ischemic-reperfusion injury (IRI) resulted in improved kidney recovery and reduced fibrosis, implying that tubular mitochondrial fission and fusion might play a role in progression of AKI to fibrosis. Moreover, Drp1 phosphorylation is also implicated in diabetic renal tubular cells (Zhan et al., 2015) and podocytes (Ayanga et al., 2016). The deletion of Drp1 selectively in podocytes of *db/db* diabetic mice leads to an attenuated diabetic phenotype, such as seen through excessive oxygen consumption (Ayanga et al., 2016).

### Mitochondria and ER Crosstalk

Eukaryotic cells contain various organelles besides the mitochondria. The functions of these individual organelles and the communication between them are essential for cell survival, proliferation, and differentiation. Intracellular transfer of vesicles enables communication between distant organelles via microtubules and actin along the cytoskeleton (Allan and Schroer, 1999; Bonifacino and Glick, 2004). Moreover, different organelles can also communicate with each other through direct contact and proximity signaling. The ER is located at the center of the membrane contact site (MCS) between organelles. Particularly, mitochondria-associated membranes (MAMs) have been intensively studied as a type of MCS formed through the ER (Fujimoto and Hayashi, 2011; Inoue et al., 2019). This machinery forms a raft-like structure rich in cholesterol and sphingolipids similar to caveola (Hayashi and Su, 2007) and enables molecular communications between ER and mitochondria via calcium, lipid synthases, inositol trisphosphate (IP3) receptors, and sarco/endoplasmic reticulum calcium-ATPases that are abundant and active on the MAM (Appenzeller-Herzog and Simmen, 2016). Notably, mitochondrial fission-promoting enzyme Mfn2 is located both in the mitochondria and partially in the ER. Furthermore, Mfn2 on the ER assembles a molecular complex with Mfn1 on the mitochondria that tightens the connection between ER and mitochondria (de Brito and Scorrano, 2008).

In the murine diabetic kidney, the MAM is significantly reduced and resembles the severity of tissue damage (Yang et al., 2019). Cultured tubular cells overexpressing the MAM-uncoupling protein (UCP), FATE-1, are resistant to high-glucose stimuli and have less cellular apoptosis (Yang et al., 2019). Although the mitochondrial-ER association has only lately been highlighted (Inoue et al., 2019; Yang et al., 2020), how this organelle crosstalk is altered in AKI or chronic kidney disease (CKD) remains largely unknown.

### Energetics

#### Glycolysis and Tricarboxylic Acid (TCA) Cycle

Glycolysis is considered the most primitive metabolic system. This process catabolizes glucose into organic acids, such as pyruvate, and produces energy for the organism to consume. Under aerobic conditions, pyruvate produced by glycolysis is transported from the cytosol to the mitochondrial matrix through an active transporter. It is next decarboxylated by the pyruvate dehydrogenase complex (PDHC) and then converted to acetyl-CoA (Semenza, 2011; Gray et al., 2014). The acetyl-CoA enters the TCA cycle, which oxidizes acetyl-CoA to produce carbon dioxide. TCA produces coenzymes as three molecules of NADH, one molecule of FADH_2_, GTP. Although carbon dioxide is excreted outside the mitochondria and no ATP is produced in the TCA cycle, the resulting coenzymes aide ATP production in the subsequent oxidative phosphorylation.

In ischemic AKI, glucose metabolism is differently altered in the cortex and the medulla of the kidney. Metabolites pyruvate and lactate are decreased in the cortex during early onset of AKI, while the TCA intermediates succinate and malate are almost unchanged (Wei et al., 2014). These observations suggest a transient decrease in the use of glucose for energy metabolism in the cortex. By contrast, the kidney medulla showed a slower decrease in glycolysis and TCA cycle activity over time. Tubular cell glucose uptake and lactate production were accelerated in fibrotic kidney resembling human CKD, implying an enhancement of aerobic glycolysis flux (Ding et al., 2017). Interestingly, induction of aerobic glycolysis led to myofibroblast activation *in vitro*. Past research has illustrated that the metabolism of the renal medulla is primarily glycolysis, whereas aerobic oxidation of substrates and gluconeogenesis is prioritized in the renal cortex (Lee et al., 1962; Lee and Peterhm, 1969). This layer-dependent renal energy preference is explained by local tissue oxygen pressure. Moreover, in the outer medulla, where slightly less oxygen is available than in the cortex, glucose fuels the mitochondria (Eveloff et al., 1980), although succinate is prioritized as an energy source (Baverel et al., 1980). In the inner medulla, glycolysis plays a more critical role in metabolizing glucose to produce energy as oxygen consumption is lower (Baverel et al., 1980).

#### Fatty Acid Transport and β-Oxidation

Most fatty acids act as energy sources for peripheral tissues once they are released into the blood through the hydrolysis of triacylglycerols in adipose tissue. Circulating triacylglycerol is degraded by lipase to produce fatty acids and glycerin. Cytosolic fatty acids are converted to acyl-CoA by acyl-CoA synthetase, which cannot pass through the IMM of the mitochondria. First, the fatty acid portion of acyl-CoA is transferred to carnitine through carnitine acyltransferase to provide acylcarnitine. Then, acylcarnitine enters the mitochondrial matrix and reacts with Coenzyme A (CoA) and returns to acyl-CoA (Bremer, 1983; Brady et al., 1993). Acyl-CoA is then decomposed into two carbon units to produce acetyl-CoA in the stepwise sequence of oxidation, hydration, oxidation, and thiol cleavage. This process, known as β-oxidation, generates ubiquinol (QH2), NADH, GTP, and a sufficient amount of ATP (Raskind and El-Chaar, 2000).

As previously mentioned, glucose is a poor energy source for respiration in the kidney cortex, which contains glomeruli and proximal tubules. The preferred fuels are short- and long-chain fatty acids and endogenous lipids, as well as ketone bodies, lactate, and some amino acids (Weidemann and Krebs, 1969; Klein et al., 1980). Conversely, chronic hyperinsulinemia enhances degradation of triglycerides in the adipocytes, thus elevating serum levels of non-esterified fatty acids. These elevated levels lead to the ectopic accumulation of lipids in organs outside of the lipid tissue, including the kidney. The excessive accumulation of lipids results in cellular damage, known as lipotoxicity (Weinberg, 2006; Ertunc and Hotamisligil, 2016; Nishi et al., 2019). For instance, fatty acids accumulated in the mitochondrial matrix are vulnerable to lipid peroxidation (Schrauwen and Hesselink, 2004; Schrauwen et al., 2010; Sergi et al., 2019), which can have lipotoxic effects on DNA, RNA, and proteins of the mitochondrial machinery, leading to organelle dysfunction. In AKI and diabetic nephropathy, β-oxidation in the mitochondria is decreased and the formation of lipid droplets inside the cell are increased, resulting in diminished ATP production (Simon and Hertig, 2015). Stimulation of proximal tubular culture cells with the fatty acid palmitate invokes the accumulation of abnormal organelles because of poor acidification of lysosomes (Yamamoto et al., 2017), even promoting lipoapoptosis (Katsoulieris et al., 2010).

#### Electron Transfer System and Mitochondrial Reactive Oxygen Species

The ETC is responsible for mitochondrial oxidative phosphorylation, which produces ATP using the oxygen-based fatty acids and pyruvate. In this process, five electron-transporting enzyme complexes (complexes I to V) on the IMM, as well as the electron transporters ubiquinone and cytochrome c, excrete protons into the intermembrane space to drive ATP synthase. Although this process is efficient, insufficient reactions can produce reactive oxygen species (ROS). Oxidative stress reduces mitochondrial and cellular function and can even cause cell death by promoting lipid peroxidation in the IMM. This lipid peroxidation can change the membrane permeability and structure (Stewart and Heales, 2003) or disrupting calcium homeostasis, particularly affecting the oxidation state of specific thiol groups in proteins (Toescu, 2005). Mitochondria possess defense systems to scavenge ROS to protect them from excess radicals. For example, superoxide dismutase 2 (SOD2) transforms superoxide anions to oxygen and hydrogen peroxide (Weisiger and Fridovich, 1973). Also, coenzyme Q10 (CoQ10) can exist in two forms: Ubiquinone, an oxidized form, that acts as an electron carrier during mitochondrial respiration; and Ubiquinol, a reduced form, that is an endogenous antioxidant (Crane, 2001). Mutations in the genes that encode the CoQ10 pathway confer an inherited mitochondriopathy with primary renal involvement (Diomedi-Camassei et al., 2007).

In chronic hypoxic kidneys of rat, proteomic analysis identified maladaptive suppression of Cu/Zn-SOD enzymes as a mediator of a cycle of oxidative stress and subsequent renal injury (Son et al., 2008). Also, mice with cisplatin-induced AKI treated with CoQ10 has less depletion of their antioxidant defense mechanisms (glutathione level and SOD activity), lipid peroxidation, and renal tissue damage (Fouad et al., 2010). The significance of mitochondrial ROS levels has also been implicated in diabetic kidney diseases (DKD). Mitochondrial superoxide and ATP production were increased in type 2 diabetic *db/db* mice in the renal cortex, compared to control mice (Sourris et al., 2012); however, the excessive renal mitochondrial hydrogen peroxide production and membrane potential seen in *db/db* mice were attenuated with CoQ10 treatment. This normalization of mitochondrial ROS generation caused by CoQ10 treatment decreases albuminuria (Sourris et al., 2012). NADPH oxidases (NOX) are the principal enzymes to generate nitrogen species and ROS, with NOX4 localized within the renal cortex and upregulated under hyperglycemic conditions within the mitochondria (Block et al., 2009). Intriguingly, pharmacological inhibition of NOX4 has potential for treatment of renal histopathology and albuminuria in *db/db* mice (Sedeek et al., 2013). Also, chemical inactivation of NOX4 protected ApoE-deficient mice treated with streptozotocin from both structural and functional kidney damage (Jha et al., 2014).

#### Electrochemical Gradient and Uncoupling

The pumping of protons into the transmembrane space results in a difference of proton concentrations between the inside and outside the IMM (i.e., electrochemical gradient). These protons can return to the matrix through the ATP synthase pump, at which uses the potential to generate ATP from adenosine diphosphate (ADP) and inorganic phosphate (Pi). The generated ATP is then transported from the mitochondria to the cytoplasm by ATP/ADP transporters and becomes the active energy source for cells. Alternatively, proton in the mitochondrial intermembrane space released by the ETC may also return to the matrix by diffusing through the IMM without involvement in ATP synthesis. This flux is known as uncoupling, and the accumulated electrochemical potential is squandered as heat (Dlaskova et al., 2006). The transporter protein UCP uses this proton gradient between membranes to generate energy for oxidative phosphorylation (Ricquier et al., 1991).

The UCP has isoforms in mammals (UCP1–5). Thermogenin (UCP1) is present only in brown adipocytes, and contributes to heat production without movement during hibernation (Palou et al., 1998). Recently, UCP1 expression was reported in the ischemic kidneys (Jia et al., 2019) and speculated to protect the organ from hypoxia, since deletion of *UCP1* worsened both ischemia or cisplatin induced AKI. Furthermore, peroxisome proliferator-activator receptor (PPAR) γ agonist treatment increased UCP1 expression, suggesting their close relationship. Elsewhere, an early work combined immunohistochemistry and polymerase chain reaction techniques to unravel the UCP2 expression in rat kidneys, specifically the epithelial cells of the proximal tubules and the medullary thick ascending loop of Henle (Balaban and Mandel, 1980). Multiple reports suggest that tubular UCP2 expression is enhanced in kidneys that are diseased. Higher expression of UCP2 is found in diabetic kidneys (Balaban and Mandel, 1980). It was demonstrated that glutamate-stimulated oxygen consumption was increased in the isolated mitochondria from diabetic animals, and could be reduced by adding guanosine diphosphate, which inhibits UCP activity. These results imply that those mitochondria have increased uncoupling due to increased UCP2 protein expression (Friederich et al., 2008). In fibrotic kidneys, the expression of UCP2 in proximal tubular epithelial cells was increased (Jiang et al., 2013), and mice deficient in UCP2 were protected from kidney fibrosis induced by unilateral ureter obstruction (UUO). Intriguingly, UCP2 causes cultured epithelial cells to transdifferentiate and release cellular matrix. Regarding other UCP isoforms, UCP3 expression is found in the epithelial cells of the renal cell carcinoma (Braun et al., 2015). Intriguingly, their loss-of-function study indicates that UCP3 in carcinoma that originated from the proximal tubular cells helps resist against hypoxia/reoxygenation injury of cancer.

### Calcium Storage

Intracellular calcium concentration is tightly regulated and plays a vital role in the signal transduction of cells. Although the ER stores the highest amount of intracellular calcium, mitochondria also have temporary capacity to store calcium, which contributes to the homeostasis of the overall intracellular calcium concentration (Brookes et al., 2004).

In kidney diseases, mitochondrial calcium uptake is impaired in tubular epithelia cells. Cation uptake in tubular cells is reduced per the cytoplasmic glutathione level in cisplatin-induced kidney damage (Kameyama and Gemba, 1991). Autosomal dominant polycystic kidney disease (ADPKD) is one of the most common monogenetic diseases, constituting approximately 5% of all kidney failure diseases (Levy and Feingold, 2000). Genes *PKD1* (The European Polycystic Kidney Disease Consortium, 1994) and *PKD2* (Mochizuki et al., 1996) encode polycystin 1 (PC1) and polycystin 2 (PC2), respectively, that are responsible for ADPKD; yet, the biological function of polycystins remains elusive and controversial (Douguet et al., 2019). Initial studies reported that PC1 and PC2 form a molecular complex configured as a calcium-permeable channel at the plasma membrane of renal tubular cells or neurons (Hanaoka et al., 2000; Delmas et al., 2004). Additionally, these PC1–PC2 complexes located in the primary cilium of kidney cells work as mechano-sensors to the intra-tubular shear stress (Nauli et al., 2003; AbouAlaiwi et al., 2009), but this mechanism has been challenged by subsequent data showing that stimulation of the primary cilium does not induce an increase in intraciliary calcium (Delling et al., 2016). Recently, researchers who focused on localization of the PC complex in MAMs discovered a novel role of these polycystins to regulate mitochondrial function (Padovano et al., 2017). PC1 interacts with the prolyl hydroxylase domain-containing protein (PHD3) to sense local oxygen pressure, and fluctuations in oxygen levels and the PHD3 activity modulate the subcellular localization and the calcium channel activity of the PC complex. PC1-deficient tubular cells had reduced oxygen consumption rate *in vitro*, consistent with a reduction in the quantity of oxidative phosphorylation performed by these cells. Thus, deficiency in the PC1 protein may mimic a relatively low oxygen pressure and lead to mitochondrial dysfunction.

### Mitophagy: Mitochondrial Quality Control

Malfunctioning and defective mitochondria are degraded through autophagy known as mitophagy. Impaired mitophagy causes the accumulation of abnormal organelles and severe mitochondrial dysfunction. The PTEN-induced kinase 1 (PINK1)–Parkin pathway is the most widely studied mechanism of mitophagy of neuron (Pickrell and Youle, 2015). Initially, both PINK1 and Parkin were known to be causative mutations in juvenile Parkinson’s disease. For example, once mitochondria are damaged and have reduced membrane potential, PINK1 accumulates on the OMM, which recruits the E3 ubiquitin ligase, Parkin, for ubiquitination of PINK1. Then, light-chain-3 (LC3) receptors accumulate and invoke the autophagosome to degrade the dysfunctional mitochondria. In addition, BCL2 Interacting Protein 3 (BNIP3) and NIX in the OMM also regulate mitophagy (Zhang and Ney, 2009). In hypoxic cells, hypoxia-inducible factor-1 (HIF-1) is stabilized and promotes expression of the target genes that eradicates damaged mitochondria (Zhang and Ney, 2008). Furthermore, FUN14 Domain Containing 1 (FUNDC1) in the OMM associates mitophagy with the LC3 receptor through its LC3-interacting region (LIR) domains under hypoxic conditions (Lv et al., 2017; Zhang et al., 2017). Under normal conditions, FUNDC1 is phosphorylated and this moiety blocks binding to LC3.

The pathogenic role of mitophagy in kidney disease still requires further investigation (Bhatia and Choi, 2019; Wang et al., 2020). Tissue damage induced by renal ischemia is prolonged in mice deficient in PINK1, PARK2, or both, representing severe mitochondrial damage and higher ROS production (Tang et al., 2018). The PINK1-Parkin pathway expression is increased in tubular cells stimulated with cisplatin. Interestingly, the silencing of PINK1 or Parkin attenuates mitophagy and promotes cell apoptosis as visualized with immunofluorescence microscopy (Zhao et al., 2017). The protective effect of PINK1/PARK2-dependent mitophagy can also be demonstrated in AKI induced by contrast media (Yang et al., 2018). The BNIP3 expression in cultured renal tubular cells is enhanced by oxygen–glucose deprivation/reperfusion. Finally, BNIP3-deficient mice demonstrate worsened renal ischemic injury due to impaired mitophagy (Tang et al., 2019). These findings suggest that the proper coordination of mitophagy is critical for protection against acute nephrotoxicity, and chronic renal fibrosis is also regulated by the PINK1–Parkin pathway in macrophages (Bhatia et al., 2019).

### Apoptosis

Apoptosis is an active and molecular programmed cell death that requires energy to occur. Oxidant stress, abundant cytokines, or hypoxia deteriorate the mitochondrial membrane potential by excreting ROS and cytochrome c from the mitochondria to trigger apoptosis through the p53 and Bcl-2 family proteins (Susnow et al., 2009; Redza-Dutordoir and Averill-Bates, 2016). Cytochrome c binds to the cytoplasmic caspase-9 and forms an aggregate that activates the caspase-9 and inducing apoptosis (Bratton and Salvesen, 2010).

In a damaged kidney, epithelial cells (Shimizu and Yamanaka, 1993) and partially podocytes (Shankland, 2006) are the principal cell types that undergo cell death. The signaling pathway underlying kidney cell apoptosis is not always p53-mediated. Cisplatin can induce Bax-mediated apoptosis in primary-cultured tubular cells isolated from mice deficient in p53 (Jiang et al., 2009), suggesting only a partial effect of p53 inhibition on cisplatin nephrotoxicity. Conversely, apoptosis of the renal cells may also be beneficial during the recovery phase, and assist in fine-tuning the number of renal cells created by balancing an exaggerated proliferative response.

## Mitochondria and Sarcopenia in CKD

### Uremic Sarcopenia

Sarcopenia is the progressive reduction of muscle weight and strength, leading to poor physical activity and quality of life, and even increasing the risk of death. Age-related muscle loss and dysfunction were initially defined as sarcopenia, whereas degradation from a chronic inflammatory or malnutritional illness is classified as secondary sarcopenia. CKD is a chronic illness that exhibits sarcopenia symptoms (Moorthi and Avin, 2017). The muscle mass is reduced in those with a greater amount of albuminuria or a lower glomerular filtration rate (GFR) (Foley et al., 2007). Importantly, CKD patients with sarcopenia show higher rates of mortality and longer hospital stays (Sinkeler et al., 2013; Pereira et al., 2015). Epidemiological evidence suggests that multiple lifestyle and clinical factors contribute to the progression of sarcopenia in CKD, including malnutrition, reduced protein intake, insufficient or deficient exercise, chronic inflammation, metabolic acidosis, atherosclerosis, and a lack of natural vitamin D (Stenvinkel and Alvestrand, 2002; Delano and Moldawer, 2006).

### Mitochondrial Dysfunction in Skeletal Muscles With Uremia

**FIGURE 2:**
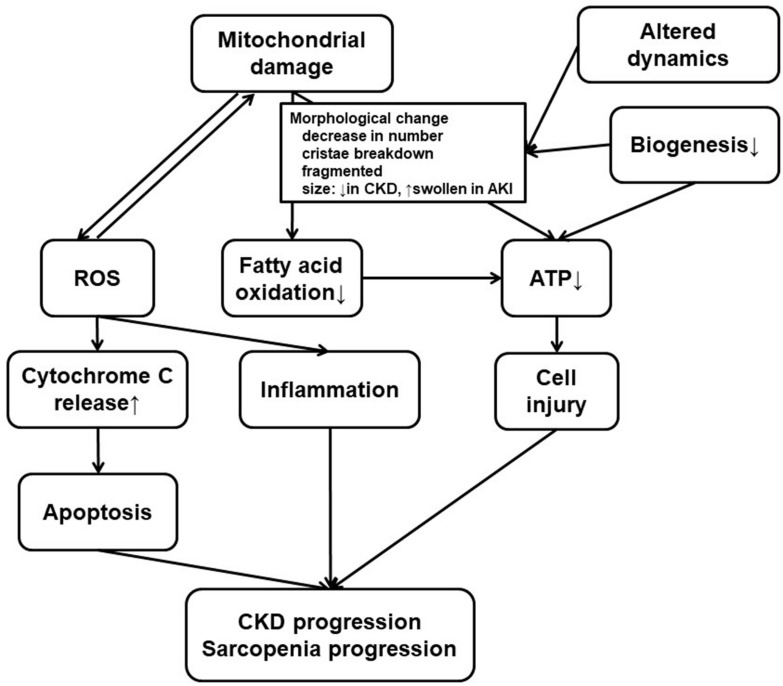
Mitochondrial dysfunction in the kidney and the skeletal muscle. Both kidney failure and sarcopenia are associated with mitochondrial damage, and there are several common findings or processes in the kidney and the skeletal muscle. Mitochondrial damage, usually accompanied with morphological change for altered dynamics and decreased biogenesis, results in ROS accumulation or deficiency in ATP production. ROS production, which can induce mitochondrial damage, promote inflammation or cytochrome C release leading to apoptosis. Low efficiency in ATP production also leads to cell injury. CKD progression or uremic sarcopenia can result from combination of these phenomena. Therapeutics for CKD have various effects on some or all of these processes. At the same time, these processes can still be a novel therapeutic target. CKD, chronic kidney disease; ROS, reactive oxygen species; ATP, adenosine triphosphate.

Several experiments indicate mitochondrial dysfunction in the skeletal muscle of patients with CKD (Gamboa et al., 2016; Sato et al., 2016; Kikuchi et al., 2019; Thome et al., 2019; Xu et al., 2020). Both mitochondrial volume density and mtDNA copy numbers were decreased in skeletal biopsy specimens sampled from kidney failure patients who underwent HD (Gamboa et al., 2016) or PD (Xu et al., 2020) (Figures 1B, 2). A recent human study assessed the phosphocreatine recovery time constant to measure mitochondrial function in the knee extensors using with ^31^P magnetic resonance spectroscopy (Gamboa et al., 2020). This study demonstrated that the phosphocreatine recovery was extended in pre-dialysis CKD as well as HD patients, compared to healthy control participants (Gamboa et al., 2020). The mitochondrial dysfunction in human skeletal muscle was also associated with poor physical activity performance when evaluated with a 6-min walk test. This result indicates that uremic sarcopenia is already progressing even before CKD advances (Nishi et al., 2020; Ryan, 2020).

Several studies have recognized the impaired role of the PDHC at a molecular level in the mitochondria of skeletal muscle in CKD sarcopenia (Sato et al., 2016; Thome et al., 2019; Xu et al., 2020). The PDHC is essential to energy production under anaerobic condition, as the enzyme converts pyruvate to acetyl-CoA for the TCA cycle. Insufficient activation of PDHC prevents TCA cycle metabolism and reduces ATP production in the mitochondria, resulting in an energy shortage in the skeletal muscle. The activity of the PDHC is regulated by the expression of several kinases and phosphatase via reversible phosphorylation. Inhibition occurs when phosphorylated at Ser232, Ser293, and Ser300 of the PDH E1-α subunit by the PDH kinases PDK-1, PDK-2, PDK-3, and PDK-4, and re-activated by dephosphorylation by the two PDH phosphatases PDP1 and PDP2. In murine C2C12 myocytes, cell cultures exposed to a uremic toxin promote glycolysis with excess antioxidative responses, leading to mitochondrial TCA cycle down-regulation and ATP shortage (Sato et al., 2016). Moreover, PDHC activity and phospho-PDH (S293) are decreased in the skeletal muscle of patients with advanced CKD, whereas PDK4 protein levels are upregulated (Xu et al., 2020). Treatment of CKD in mice with dichloroacetate to activate PDHC improved the treadmill running test distance (Tamaki et al., 2014). The uremic condition does not impair the overall activity of mitochondrial enzymes. Isolated mitochondria from murine skeletal muscle had impaired malate and glutamate dehydrogenases, as well as ETC complexes III and IV, by several uremic metabolites (Thome et al., 2019). Treatment with 5-aminolevulinic acid to transport electrons in the mitochondrial ETC also increases the skeletal muscle weight and the mitochondrial amount, thereby improving physical activity (Fujii et al., 2017). Altogether, the reinforcement of muscle mitochondria function serves a potential strategy for eradicating uremic sarcopenia.

## Drugs

Almost no treatments have been approved for slowing or reversing CKD progression, although various treatments for CKD and its complications are currently being trialed. These treatment approaches may potentially have beneficial effects, such as relieving renal fibrosis (a hallmark of CKD) and minimizing AKI in patients, who are predisposed to the development and progression of CKD. This chapter discusses the various therapeutic agents and potentially protective agents for CKD with a focus on the pharmacological effects on the mitochondria in the kidney or skeletal muscle.

### Erythropoietin

Erythropoietin (EPO) analogs are a major therapeutic approach to treating anemia from EPO deficiency in CKD. Erythropoietin replenishment has been reported to slow CKD progression (Gouva et al., 2004), as EPO may provide renoprotection from some factors related to the mitochondria. Erythropoietin ameliorates lipopolysaccharide-induced AKI (Stoyanoff et al., 2014). Renoprotection is promoted via an anti-apoptotic effect from the expression of the EPO receptor, the reduction of the Bax/Bcl-XL ratio, the inhibition of cytochrome-c release into the cytosol, and the decrease of active caspase-3 expression. EPO treatment also reduces renal fibrosis in UUO model rats, and downregulates Drp1 overexpression to reduce mitochondria fission (Zhao et al., 2015).

Patients with CKD develop impaired mitochondrial energetics associated with the disease severity in skeletal muscle (Kestenbaum et al., 2020). CKD mice with muscle atrophy have decreased mitochondrial activity and amount, metabolism related to AMP-activated protein kinase (AMPK) phosphorylation and *Pgc1*α gene expression (Tamaki et al., 2014). The EPO receptor expression can be seen in skeletal muscle (Lamon and Russell, 2013), and EPO treatment increases the PGC-1α protein and gene expression in combination with exercise (Pin et al., 2015). Elevated EPO signaling leads to the activation of mitochondrial biogenesis and metabolism as indicated by increased AMPK phosphorylation, PGC1α expression, and oxygen consumption rate in both *in vitro* and *in vivo* models (Wang et al., 2013). Thus, EPO treatment may alleviate mitochondrial dysfunction in skeletal muscle in CKD and improve muscle amount and performance.

### HIF-PH Inhibitor

Hypoxia-inducible factor prolyl hydroxylase (HIF-PH) inhibitors are another emerging therapeutics for renal anemia, as these inhibitors have already been effective in treating patients with or without dialysis (Chen et al., 2019a,b). HIF-PHs stimulates EPO production in the kidney via the activation of the HIF pathway. The agents exert non-hematopoietic effects, as HIF up-regulates the transcription of more than 100 genes involved in hypoxic adaptation. Moreover, HIF-1α regulates cellular metabolism against oxidative phosphorylation via LDHA, PDK, and COX4-2 upregulation and encourages mitochondrial autophagy through BNIP upregulation, resulting in the optimized efficiency of mitochondrial respiration and ROS production (Semenza et al., 1996; Kim et al., 2006; Papandreou et al., 2006; Fukuda et al., 2007; Zhang et al., 2008; Bellot et al., 2009). Moreover, HIF-1α is crucial in preventing mitochondrial dysfunction and apoptosis under hypoxic conditions. Cobalt chloride salt is a classical HIF-PH inhibitor that protects against cisplatin-induced kidney injury in mice (Tanaka et al., 2005). The emerging HIF-PH inhibitor enarodustat also suppresses mitochondrial respiration in HK-2 tubular cells and changes energy metabolism in early DKD mice (Hasegawa et al., 2020).

HIF-1α plays a part in regulation of skeletal muscle function, since a skeletal muscle-specific HIF-1α knockouts in mice have increased mitochondrial activity with higher citrate synthase activity and oxidative metabolism as well as slight increases in the mitochondrial amount and endurance capacity (Mason et al., 2004). Moreover, PHD1 is also associated with mitochondrial changes in skeletal muscle (Aragones et al., 2008). The silencing of PHD1 in myofiber cells decreases oxidative metabolism and less mitochondrial ultrastructural changes compared to ischemia-exhibiting control myofibers with swollen mitochondria, inner lucency, and fractured cristae (Aragones et al., 2008). HIF-PH inhibitors have also been reported to reduce the levels of ROS with glycolytic metabolic shift and increase cell viability in renal proximal tubule cells (Ito et al., 2020) and neuronal cells with glutamate-induced oxytosis (Neitemeier et al., 2016). HIF-PH inhibitor MK-8617 ameliorates myopathy in 5/6 nephrectomy CKD mice and corrects abnormalities in the mitochondrial number and size in skeletal muscle (Qian et al., 2019). Future HIF-PH inhibitors as novel HIF stabilizers may relieve uremic sarcopenia, but they require further investigation.

### Nrf2 Activator

The progression of CKD leads to oxidative stress and impaired antioxidant capacity that are associated with the impairment of Nrf2 activity (Ruiz et al., 2013). Bardoxolone methyl, an Nrf2-activating triterpenoid, has been reported to improve renal function in DKD in humans (de Zeeuw et al., 2013), although this drug is not yet approved for patient use. Bardoxolone methyl improved the estimated GFR (eGFR) above baseline in CKD/type 2 diabetes patients in a BEAM randomized, placebo-controlled, 52 week trial (Pergola et al., 2011); however, this trial was prematurely terminated due to as a result of an increased rate of cardiovascular events leading to hospitalization or death in the treatment group because of heart failure (de Zeeuw et al., 2013). Thereafter, the efficacy on DKD was evaluated in a TSUBAKI clinical study in Japan that payed careful attention to cardiac events by excluding patients with risk factors for volume overload or prior history of heart failure (Nangaku et al., 2020). Nrf2 activation also relieves renal injury in non-DKD model mice, and the Nrf2-activating triterpenoid CDDO-imidazolide seems to protect the kidney from ischemia-reperfusion injury by decreasing ROS production in mice (Liu et al., 2014). Another Nrf2 activator, dihydro-CDDO-trifluoroethyl amide (dh404), alleviates proteinuria-induced tubular damage by stopping mitochondrial structural changes, such as decreasing size, number, and breakdown of the cristae structure of mitochondria *in vivo* (Nagasu et al., 2019). Moreover, activator dh404 also decreased mitochondrial ROS and the preservation of mitochondrial membrane potential *in vitro* (Nagasu et al., 2019).

Nrf2 is important for maintaining mitochondrial function, muscle mitohormesis, and oxidative stress defense in skeletal muscle (Coleman et al., 2018; Kitaoka et al., 2019). Moreover, Nrf2 is involved in uremic sarcopenia, which has reduced skeletal muscle mitochondrial mass and gene expression related to mitochondrial biogenesis, including Nrf2 (Liu et al., 2019; Watson et al., 2020). Genetic Nrf2 activation in skeletal muscle improves endurance capacity and increased oxygen consumption without altering mtDNA content (Uruno et al., 2016). Treatment with Nrf2-activating compounds also increases running endurance in rodents (Uruno et al., 2016). Altogether, these results indicate that Nrf2 activators could be effective therapeutic against sarcopenia in CKD patients and require further investigation.

### SGLT2 Inhibitor

Recent research has focused on sodium–glucose transporter 2 (SGLT2) inhibitors as a beneficial treatment for DKD. The CREDENCE trial assessed the effects of inhibitor canagliflozin on renal conditions in patients with type 2 diabetes and albuminuric CKD (Perkovic et al., 2019). Canagliflozin had favorable results with a 30% risk reduction in the composite outcome of kidney failure (dialysis, transplantation, or a sustained estimated GFR of <15 ml per minute per 1.73 m^2^), a doubling of the serum creatinine level, or death from renal or cardiovascular causes, thereby resulting in early cessation of the trial (Perkovic et al., 2019). Similarly, early cessation of the DAPA-CKD trial was also announced by AstraZeneka (Heerspink et al., 2020), whereas their EMPA-KIDNEY trial is still under way^1^. There are various mechanisms of renoprotection exhibited by the SGLT2 inhibitors, including a decrease in blood glucose levels, tubuloglomerular feedback, upregulation of HIF and EPO, and subsequent hematocrit elevation and oxygen supply (Vallon and Thomson, 2020). The inhibitor ipragliflozin protects tubular cells in the high-fat diet-fed mice (Takagi et al., 2018). These mice show no longer abnormal mitochondrial fission associated with increased oxidative stress, lower gene expression of Opa1 and Mfn2, and higher expression of Drp1 (Takagi et al., 2018). Ipragliflozin also reduces ROS and mitochondrial dysfunction in tubular epithelial cells and glomerular podocytes in diabetic *db/db* mice (Kamezaki et al., 2018).

SGLT2 inhibitors have also been reported to have protective effects against muscle atrophy and lowered exercise performance (Hirata et al., 2019). Empaglifrozin improves symptoms for diabetic sarcopenia in hyperglycemic Akita mice, though it is unclear whether there are other factors beyond the anti-diabetic effects and improving muscle mass (Hirata et al., 2019). Empagliflozin also restores lowered exercise capacity in a murine heart failure model (Nambu et al., 2020). The drug increases endurance capacity, but not muscle weight or muscle strength, by restoring mitochondrial fatty acid oxidation in skeletal muscle (Nambu et al., 2020).

### AST-120

AST-120 is an agent that inhibits the accumulation of uremic toxins and is often prescribed to CKD patients to slow the progression of renal failure. The efficacy of AST-120 to slow down CKD progression is controversial, as various clinical trials have failed to show renoprotective effects (Schulman et al., 2015; Cha et al., 2016); however, the drug remains a standard method of treatment for CKD patients.

AST-120 may have beneficial effects on muscles since uremic toxins are harmful to them. AST-120 may improve mitochondrial status by reducing the accumulation of indoxyl sulfate, which induces mitochondrial dysfunction and ATP shortage in muscle cells (Sato et al., 2016). As for *in vivo* models, AST-120 administration improves running endurance reduced in subtotal nephrectomy mice, and attenuates harmful changes, such as down-regulated citrate synthase activity, decreased expression of mitochondrial biogenesis genes like *Pgc1*α, and increased superoxide production (Nishikawa et al., 2015). There are no significant reports on the clinical usage of AST-120 against uremic sarcopenia, so further studies are required to indicate clinical efficacy.

### Carnitine

Patients with pre-dialysis CKD have higher plasma L-carnitine levels than healthy individuals (Rodriguez-Segade et al., 1986; Guarnieri et al., 1987). Nevertheless, hemodialysis patients show low plasma and muscle L-carnitine levels that correlate with the dialysis vintage (Sakurauchi et al., 1998; Debska et al., 2000; Evans, 2003).

Carnitine deficiency is associated with EPO-resistant anemia, intradialytic hypotension, cardiomyopathy, and skeletal muscle dysfunction (Karpati et al., 1975). Therefore, L-carnitine supplementation is recommended to relieve such problems (Eknoyan et al., 2003). There is no firm conclusion regarding the clinical efficacy of L-carnitine on skeletal muscle (Hurot et al., 2002), although some trials have shown improvement in muscle volume, strength, and maximal oxygen consumption (Ahmad et al., 1990; Siami et al., 1991). In animal models, L-carnitine improved endurance capacity lowered in CKD mice, normalized PGC-1α expression, and reduced a blunt reduction in type I muscle fibers seen in untreated controls (Enoki et al., 2017).

## Future Directions

Although mitochondrial dysfunction has been involved in various pathologies, including CKD and sarcopenia, clinical impact of this organelle dysfunction in patients with CKD has not been fully explored. This article reviewed essential mitochondrial functions, mitochondrial changes in CKD and sarcopenia conditions, and the effects of emerging therapeutics on the kidney and skeletal muscle. A comprehensive understanding of mitochondrial physiology is critical for understanding the pathogenesis of kidney diseases and muscle wasting. Furthermore, therapeutic strategies against mitochondrial dysfunctions could lead to drastic progress in the treatment and regression of CKD or sarcopenia.

## Author Contributions

KT wrote the original manuscript. HN and RI revised the manuscript. All authors contributed to the article and approved the submitted version.

## Conflict of Interest

The authors declare that the research was conducted in the absence of any commercial or financial relationships that could be construed as a potential conflict of interest.

## Footnotes

1 www.empakidney.org; accessed April 24, 2020
